# Extracting film thickness and optical constants from spectrophotometric data by evolutionary optimization

**DOI:** 10.1371/journal.pone.0276555

**Published:** 2022-11-30

**Authors:** Rajdeep Dutta, Siyu Isaac Parker Tian, Zhe Liu, Madhavkrishnan Lakshminarayanan, Selvaraj Venkataraj, Yuanhang Cheng, Daniil Bash, Vijila Chellappan, Tonio Buonassisi, Senthilnath Jayavelu

**Affiliations:** 1 Institute for Infocomm Research (I^2^R), Agency for Science, Technology and Research (A*STAR), Singapore, Singapore; 2 Low Energy Electronic Systems (LEES), Singapore-MIT Alliance for Research and Technology (SMART), Singapore, Singapore; 3 Solar Energy Research Institute of Singapore (SERIS), National University of Singapore, Singapore, Singapore; 4 Department of Mechanical Engineering, Massachusetts Institute of Technology, Cambridge, MA, United States of America; 5 Institute of Materials Research and Engineering (IMRE), Agency for Science, Technology and Research (A*STAR), Singapore, Singapore; 6 Artificial Intelligence, Analytics And Informatics (AI^3^), A*STAR, Singapore, Singapore; Universiti Brunei Darussalam, BRUNEI DARUSSALAM

## Abstract

In this paper, we propose a simple and elegant method to extract the thickness and the optical constants of various films from the reflectance and transmittance spectra in the wavelength range of 350 − 1000 nm. The underlying inverse problem is posed here as an optimization problem. To find unique solutions to this problem, we adopt an evolutionary optimization approach that drives a population of candidate solutions towards the global optimum. An ensemble of Tauc-Lorentz Oscillators (TLOs) and an ensemble of Gaussian Oscillators (GOs), are leveraged to compute the reflectance and transmittance spectra for different candidate thickness values and refractive index profiles. This model-based optimization is solved using two efficient evolutionary algorithms (EAs), namely genetic algorithm (GA) and covariance matrix adaptation evolution strategy (CMAES), such that the resulting spectra simultaneously fit all the given data points in the admissible wavelength range. Numerical results validate the effectiveness of the proposed approach in estimating the optical parameters of interest.

## Introduction

The determination of a thin-film’s thickness and complex refractive index over a broad spectral range is carried out using either ellipsometric or spectrophotometric analysis [1, 2]. *Ellipsometry* measures how the polarization of a light beam changes on reflection from a surface, whereas *Spectrophotometry* measures reflectance and/or transmittance of light through thin-films and substrates as function(s) of wavelength. The spectral information, reflectance and/or transmittance data, are fed to a numerical solver for extracting thickness and refractive index and/or extinction coefficient [3, 4]. The information on film thickness and optical constants play a major role in selecting suitable materials or processes for different applications such as solar cells, sensors, displays and window coatings. For example, the optical characterization of perovskite thin-films is a key requirement in photovoltaic device design [5, 6]. To determine the complex refractive indices of CsPbBr_3_ thin-films [5], researchers have used variable-angle spectroscopic ellipsometry measurements and described the associated bulk planar CsPbBr_3_ layer with Tauc-Lorentz and Gaussian oscillator models for VASE fitting technique. For optical analysis of semitransparent and opaque solar cells, the complex refractive indices of CH_3_NH_3_PbI_3_ films can be determined by using a combination of variable-angle spectroscopic ellipsometry and spectrophotometry data [6]. Film thickness influences micro-scale physics of electron mobility in thin-film transistors [7] to macro-scale physics of operating characteristics of solar cells [5]. Thickness variations by adjusting the catalyst concentration or by changing the heat-treatment process can be leveraged to shift the reflection band of a film from narrow to broad wavelength region, which benefits in fabricating dielectric reflectors for solar cells and bandpass filters for optical instruments [8]. Moreover, complex refractive index profiles and film thickness are crucial in designing appropriate polymers to produce effective lenses and ultraviolet (UV)-absorbing coatings [9, 10].

Computationally, it is not trivial to achieve accurate estimates of all the optical parameters together [11, 12], since the inverse problem of retrieving the optical characteristics of a film from spectral information is highly non-linear and often ill-conditioned. Accurate measurement of thickness using experimental methods is a time-consuming process in practice [13, 14]. Usually, the most accurate thickness is attained while compromising accuracy of the estimated refractive index [15, 16]. Moreover, different methods perform well in different spectral ranges [16, 17]. Two inherent difficulties in an inverse photometric problem are: (a) *missing information* induced by the experimental uncertainty or film imperfections [12], and (b) *multi-solution predicament* due to the fact that the measured intensities might be compatible with other plausible combinations of optical constants [18]. To dissolve the ambiguity in the parameter extraction process, previous researchers [16] exploited reflectance and transmittance spectral measurements at two distinct incident angles of light. In [19], researchers established that multiple incidence angles are not necessary for the optical analysis of a single layer, though it is crucial in analyzing multi-layer stacks with a global optimization algorithm. Thus, there emerges a need of developing efficient methods for rapid and accurate determination of the thickness and the optical constants of thin-films.

Several commercial packages of thin-film optical software, such as TFCalc, Filmwizard, Optilayer, and Essential McLeod, provide advanced modelling for optical dispersion laws and use fitting methods to determine the thickness and complex refractive index [15]. However, these packages cannot always find satisfactory solutions, especially when the initial candidate is far away from the desired solution. Due to this fact, previous researchers found the accuracy of estimates concerning in case of a strongly absorbing material like silicon in the visible range [15]. In order to extract the complex dielectric functions of different types of samples like thin-films and anisotropic crystals from optical spectra, RefFIT [20] offers a variational Kramers-Kronig constrained fitting approach that fits all the measured spectral data points simultaneously, which requires minimal readaptation in different experimental situations. A recent development, RefDex [4, 21] provided an interactive fitting procedure to extract the complex refractive index of a film with known thickness from reflectance and transmittance data. This inverse problem is not unique and there exist multiple solutions that minimize the same loss function [4], which mandates imposing physical constraints to determine meaningful solutions. The major concern is that the above-mentioned software-packages do not offer much flexibility on the choice of parameters, such as the number of oscillators and the bounds on model coefficients, involved in their respective optimization procedures. For instance, OptiChar allows an user to set lower and upper limits on the thickness, the refractive index, and the extinction coefficient, and it offers a variety of models like normal, anomal or arbitrary dispersion, and Sellmeier [15]; however, the number of variables and the optimization algorithm used in the estimation process remain unknown to an user [15].

The refractive index and extinction coefficient profiles can be substantially different for various materials [22, 23]. For instance, consider some of the metal-oxides; the complex refractive indices of the iron-oxides, Hematite and Magnetite, exhibit more peaks and valleys (optical transitions) than that of the copper oxides [24]. Due to this fact, it is difficult for a supervised learning based estimation approach to adapt to a variety of materials [25]. Recently, a machine learning model has been developed to predict the correlation between spectral data and thickness; however, this approach was suitable for thickness characterization of dielectric materials but not materials with high extinction coefficients (like titanium nitride: TiN) [26]. To attain generality, there arises a need for supervised learning along with knowledge transfer techniques. On the contrary, an evolutionary optimization approach has potential to offer flexible solutions through swarm-based efficient search space exploration [27], while circumventing intense data curation and training requirements involved in traditional supervised learning techniques [26]. Hence, in the present work, we pose the underlying inverse problem as an optimization problem and solve it with evolutionary algorithms [27].

Earlier studies explored optimization-based fitting procedures to extract optical constants using spectrophotometric data. Woollam et al. [28, 29] developed a solver that allowed sequential addition of various optical models to minimize fitting errors for extracting refractive index and thickness. A local optimizer, Levenberg-Marquardt (LM) algorithm [30], was utilized in their approach, which gradually improved the solution accuracy. The optimizer starts from an initial guess and moves it to a feasible local minimum until another model addition becomes necessary for further reduction in the fitting error. Swarm-based evolutionary algorithms (EAs) are robust and good at finding the global optimum even for high-dimensional problems [31–33], whereas single point based gradient descent variant algorithms face challenges due to local entrapment and require good initial guesses to reach the global optimum [30, 34]. Moreover, multiple local search procedures from different starting points had been utilized in the Clustering Global Optimization (CGO) algorithm to estimate film thickness and optical constants from spectrophotometric data [15, 35]. The estimation accuracy of CGO was good for *SiO*_2_ and *Ta*_2_*O*_5_ films, although it’s efficiency depends on a balanced choice of the starting intervals [35]. EAs have also been exploited to determine the optical parameters of interest [36]. Genetic algorithm (GA) and simulated annealing (SA) have been exploited in the ellipsometric evaluations, where the traditional gradient-based LM method faces difficulty in tackling the related hilly error surfaces [37]. Gao et al. [15] applied GA and SA on an ensemble of Tauc-Lorentz oscillators to extract the real and imaginary parts of complex refractive index of a film with known thickness from reflectance and transmittance spectra. However, their approach required a large population to determine the desired inverse solutions. In EAs, a large population size enhances the search space exploration and helps in avoiding local optima at the cost of computational overhead.

The present work aims to determine various thin-films’ complex refractive indices and thickness values with the help of EAs, such as GA and CMAES. Recently, covariance matrix adaptation evolution strategy (CMAES) has proved to be reliable in solving deterministic and stochastic global optimization problems even with a small population size due to the attributes like step-size adaptation, noise effect reduction, and invariance under coordinate systems [27]. Moreover, CMAES exhibited efficient performance on high-dimensional and ill-conditioned optimization problems by utilizing an isotropic (rotation-invariant) evolution path [38]. Further, the performance of the proposed method is validated on metal-oxide and perovskite films, and its computational effectiveness is justified in comparison with the existing methods. The related optical dispersion models and the proposed optimization procedure to solve the addressed inverse problem are described in *Physics to Mathematics* section. The different types of data used in the present study are illustrated in *Data Curation* section. The achieved inverse solutions, thickness values and optical constants, of different films are presented in the *Results* section along with analysis and discussion. Finally, the benefits of the proposed approach are summarized in the *Conclusion*.

## Physics to mathematics

In our spectrophotometric analysis, the optical parameters to be extracted are: thickness (*d*), refractive index (*n*), and extinction coefficient (*k*), and the measured data are: reflectance (*R*) and transmittance (*T*) spectra. The film thickness (*d*) is a scalar quantity, and the complex refractive index (*n* + *ik*) is composed of real and imaginary constituents as functions of wavelengths. The real part of a complex refractive index (*n*) describes the propagation velocity of the incident light within the film material, and the imaginary part of it (*k*) concerns about how much of the light gets absorbed in the medium. This study aims to solve the inverse problem of determining {*d*, *n*, *k*} from {*R*, *T*}. In the following, we first explain the use of optical oscillator models to emulate complex refractive index and then dive into the problem formulation.

### Forward calculations via oscillator models

The complex dielectric function, *ϵ*(*ω*) = *ϵ*_1_(*ω*) + *i* *ϵ*_2_(*ω*), is analytic in the upper half of the complex *ω* plane [23, 39], where $\omega =\frac{c}{\lambda }$ denotes the frequency of the incident light with λ being its wavelength and *c* being the speed of light in the air. The associated photon energy is represented by *E* = *hω*; *h* is Planck’s constant. The analytic behavior of *ϵ*(*ω*) stems from the principle of causality [23, 40]. Consequently, the imaginary and real parts of it are interconnected by the Kramers-Kronig relation [40, 41]. Using this relation [39], one can determine *ϵ*_1_ from *ϵ*_2_ as follows.

$$\begin{array}{c} \epsilon_{1}\left(\omega \right)=\epsilon_{1}\left(\infty \right)+\frac{2}{\pi }P\int_{0}^{\infty }\frac{\Omega \epsilon_{2}\left(\Omega \right)}{\Omega^{2}-\omega^{2}}d\Omega \;, \end{array}$$ (1)

where *P* denotes Cauchy’s principle value integral [23]. The complex dielectric function and complex refractive index are related by: *ϵ*_1_ + *iϵ*_2_ = (*n* + *ik*)^2^, which leads to

$$\begin{array}{c} \epsilon_{1}\left(\omega \right)=n^{2}\left(\omega \right)-k^{2}\left(\omega \right)\;\;\&\;\;\epsilon_{2}\left(\omega \right)=2n\left(\omega \right)\times k\left(\omega \right)\;. \end{array}$$ (2)

Substituting Eq (2) into Eq (1), unfolds the dispersion relation [23, 42] between the real (*n*) and imaginary (*k*) parts of a complex refractive index as

$$\begin{array}{c} n\left(\omega \right)=n\left(\infty \right)+\frac{2}{\pi }P\int_{0}^{\infty }\frac{k(\Omega )}{\Omega^{2}-\omega^{2}}d\Omega \;. \end{array}$$ (3)

To accurately evaluate *n*(*ω*) from *k*(*ω*), the above integration (3) requires to be solved for all frequencies ranging from zero to infinity [23, 40]. However, from an experimental perspective, it is only feasible to address a finite range of frequencies. A numerical integration with such a limited spectral range gives erroneous results [42]. Instead, if the functional form of *k*(*ω*) is known for all frequencies, then the functional form of *n*(*ω*) can be determined elegantly [23]. Therefore, oscillator models are utilized to generate tractable continuous function approximations. In this research, Tauc-Lorentz and Gaussian oscillator models are taken into account, as described below.

**Ensemble of Tauc-Lorentz Oscillators:** Consider a material with the complex dielectric function: *ϵ* = *ϵ*_1_ + *iϵ*_2_. According to an ensemble of *N* Tauc-Lorentz (TL) oscillators [15], the imaginary part of the complex dielectric function can be expressed as

$$\begin{array}{c} \epsilon_{2}\left(E=h\omega \right)=\{\begin{array}{c} \sum_{i=1}^{N}\frac{A_{i}E_{0i}C_{i}{\left(E-E_{g}\right)}^{2}}{{\left(E^{2}-E_{g}^{2}\right)}^{2}+C_{i}^{2}E^{2}}\frac{1}{E}=\sum_{i=1}^{N}\frac{A_{i}\omega_{0i}C_{i}{\left(\omega -\omega_{g}\right)}^{2}}{{\left(\omega^{2}-\omega_{g}^{2}\right)}^{2}+C_{i}^{2}\omega^{2}}\frac{1}{\omega }\;\;\;\text{for}\;\;E>E_{g}\\ 0\;\;\;\;\text{for}\;\;E\leq E_{g}\;, \end{array} \end{array}$$ (4)

where *E*_0*i*_, *E*_*g*_, *C*_*i*_, *A*_*i*_ represent the peak transition energy, the band gap energy, the broadening parameter, and the factor involving optical transition matrix elements for the *i*’th TL oscillator, respectively; *ω*_*g*_ and *ω*_0*i*_ are the respective frequencies corresponding to the energies *E*_*g*_ and *E*_0*i*_. To calculate *ϵ*_1_ from *ϵ*_2_, let us now recall the Kramers-Kronig relation (1). In practice, the lower limit of the integral in Eq (1) is chosen as *ω*_*g*_ instead of 0 because the Tauc-Lorentz model requires *ϵ*_2_ to be zero for photon energies below the band gap [39]. Note that *ϵ*_1_(∞)>1 is a high frequency dielectric constant to prevent *ϵ*_1_ → 0 when *E* < *E*_*g*_.

The formulation of these optical functions was first proposed by Forouhi and Bloomer for amorphous semiconductors and insulators [23], and later, extended for crystalline semiconductors and metals [40]. According to the seminal work by Jellison and Modine [39], *ϵ*_1_(*ω*) can be derived from Eq (1) by exploiting the continuous approximation (4) of *ϵ*_2_(*ω*). These *ϵ*_1_ and *ϵ*_2_ are then used to solve Eq (2) for all *ω*, so that *n* and *k* can be deduced as a function of (3*N* + 3) parameters, where 3*N* parameters come from (*E*_0*i*_, *C*_*i*_, *A*_*i*_) for *i* ∈ [1, *N*] and the rest three parameters are: *d*, *ω*_*g*_, and *ϵ*_1_(∞). The number of decision variables involved in optimizing an TL ensemble model are: (3*N* + 3).

**Ensemble of Gaussian Oscillators:** The imaginary r-index profile according to an ensemble of Gaussian oscillators, is given by

$$\begin{array}{c} k\left(\omega \right)=\sum_{i=1}^{N}A_{i}{\text{exp}}^{\frac{-{\left(w-\mu_{i}\right)}^{2}}{2\sigma_{i}^{2}}}=\sum_{i=1}^{N}A_{i}G_{s}\left(W_{i}\right)\;, \end{array}$$ (5)

where $G_{s}\left(W_{i}\right)={\text{exp}}^{-W_{i}^{2}};\;W_{i}=\frac{\left(w-\mu_{i}\right)}{\sqrt{2}\sigma_{i}}$ stands for the *i*^*th*^*Gaussian component* with mean *μ*_*i*_ and variance *σ*_*i*_, and *A*_*i*_ is the corresponding coefficient for all *i* = 1, 2, …, *N* [43]. Next, *n*(*ω*) is determined by applying the Kramers-Kronig integration (3) to the continuous approximation (5) of *k*(*ω*), which takes shape as

$$\begin{array}{cc} & n\left(\omega \right)=n\left(\infty \right)+\frac{2}{\sqrt{\pi }}\sum_{i=1}^{N}A_{i}{\text{exp}}^{-W_{i}^{2}}\int_{0}^{-W_{i}}{\text{exp}}^{x^{2}}dx\;, \end{array}$$ (6)

$$\begin{array}{cc} & \text{or,}\;\;n\left(\omega \right)=n\left(\infty \right)+\sum_{i=1}^{N}A_{i}G_{s}\left(W_{i}\right)\times \text{Erfi}\left(-W_{i}\right)\;, \end{array}$$ (7)

where $\text{Erfi}\left(-W_{i}\right)=\frac{2}{\sqrt{\pi }}\int_{0}^{-W_{i}}{\text{exp}}^{x^{2}}dx$ represents the *i*^*th*^*imaginary error function* [44]; *n*(∞) = 1 refers to *k*(∞) = 0 and *n*(∞)>1 refers to *k*(∞)≠0 [40, 43]. Here, the Gaussian distributions are utilized to retrieve *n* and *k* directly instead of deriving them from *ϵ*_1_ and *ϵ*_2_. An ensemble of Gaussian oscillators (GO) composed of *N* Gaussian distributions, deals with 3*N* parameters as *A*_*i*_, *μ*_*i*_, *σ*_*i*_ for *i* ∈ [1, *N*] and two more parameters as *n* and *d*. Thus, the total number of decision variables involved in optimizing an GO ensemble model are: (3*N* + 2).

Note that the above optical constants, *n*(*ω*) and *k*(*ω*), are expressed as *n*(λ) and *k*(λ), respectively, during the numerical implementation. Since $\omega =\frac{c}{\lambda }$, the order of (*n*, *k*) sequences gets reversed when the independent variable is changed from *ω* to λ. The forward calculation of {*R*_*calc*_(λ), *T*_*calc*_(λ)} for a tuple {*d*, *k*(λ), *n*(λ)} is carried out by the transfer-matrix method [17]. The transfer-matrix method is used to calculate the forward and backward propagating electric fields in smooth homogeneous films, which relies on the superposition of the induced electric fields. The overall transfer matrix is obtained by multiplying a matrix that quantifies the change in field due to the light waves propagating through an interface (air-to-film) with another matrix that quantifies the change in field due to the same waves propagating within a layer (film).

### Inverse problem formulation

**Fig 1:**
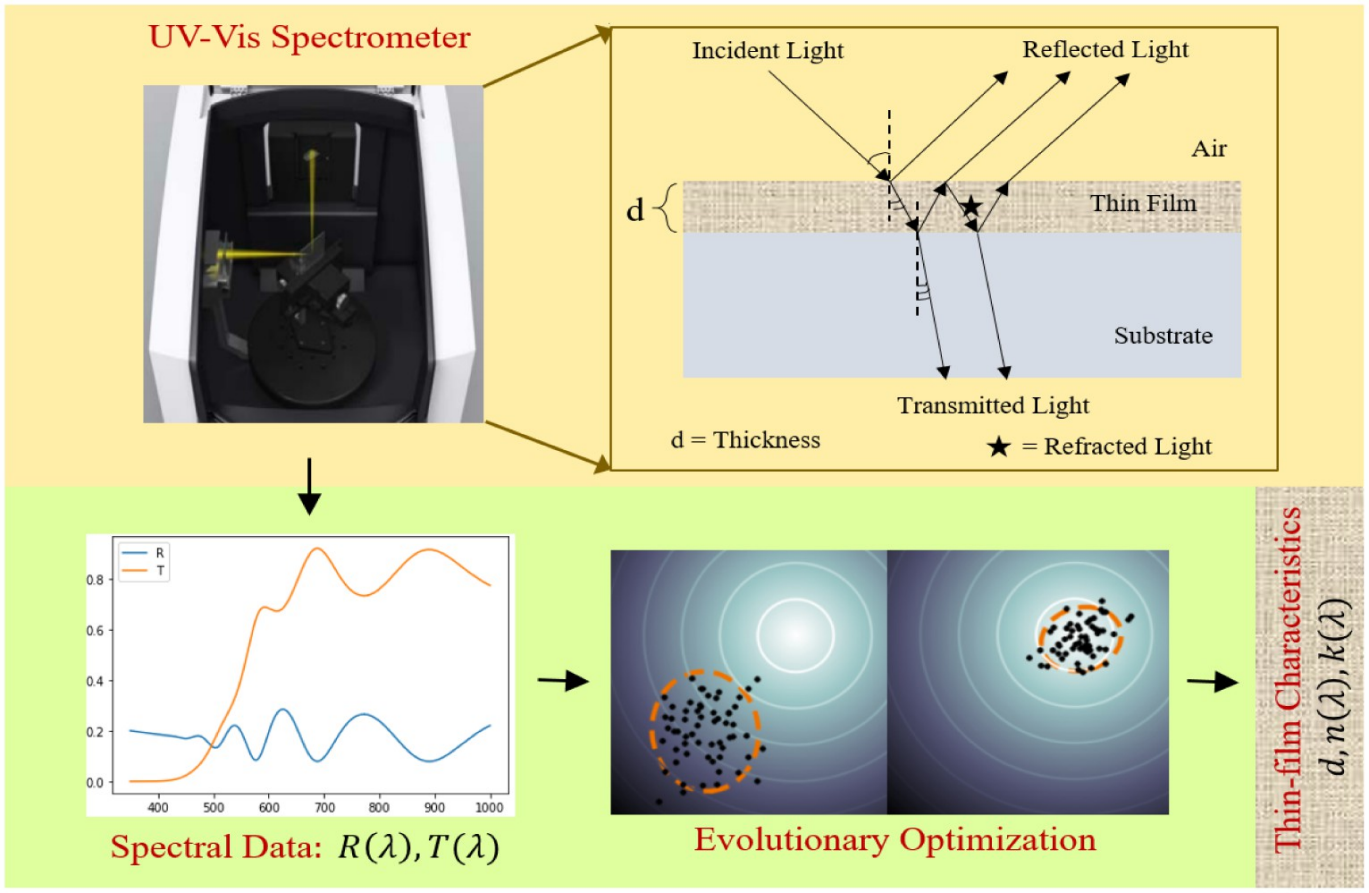
A flow diagram of the forward and inverse processes: (a) The forward process (yellow panel) involves an UV-Vis-NIR Spectrometer to measure the reflectance & transmittance {*R*(λ), *T*(λ)} resulting from a film of thickness *d* *nm* and complex refractive index: *n*(λ) + *ik*(λ), (b) The inverse problem (green panel) is to find the desired optical parameters {*d*, *n*(λ), *k*(λ)} from the measured data {*R*(λ), *T*(λ)}.

We now explain how the underlying inverse problem is posed as an optimization problem. An overview of the associated forward and inverse processes is depicted in Fig 1.

**Optimization problem.** The optimization problem is defined as

$$\begin{array}{c} \underset{d,k(\lambda ),n(\lambda )}{\text{min}}L=\sum_{\lambda =\lambda_{lb}}^{\lambda_{ub}}{\left\{R_{meas}\left(\lambda \right)-R_{calc}\left(\lambda \right)\right\}}^{2}+{\left\{T_{meas}\left(\lambda \right)-T_{calc}\left(\lambda \right)\right\}}^{2}\;. \end{array}$$ (8)

In Eq (8), *L* is the total loss containing differences in the experimentally measured reflectance and transmittance data {*R*_*meas*_, *T*_*meas*_} from their theoretically calculated values {*R*_*calc*_, *T*_*calc*_}, for all wavelengths. The admissible range of the wavelength, i.e. {λ_*lb*_, λ_*ub*_}, depends on the experimental infrastructure. The decision variables associated with the optimization problem, are: thickness *d*, real r-index profile *n*(λ), and imaginary r-index profile *k*(λ). Thickness candidates are directly passed to the solver, whereas *n* and *k* candidates are passed in terms of the oscillator model parameters. The evaluation of {*R*_*calc*_(λ), *T*_*calc*_(λ)} for a candidate solution {*d*, *n*(λ), *k*(λ)} has been discussed in the preceding section.

In order to make the optimization process computationally efficient, we generate candidate solutions within a search space restricted by an intrinsic correlation [23, 42] between the optical parameters of interest. The thickness candidates are chosen from a reasonable range of scalars. The imaginary and real refractive index candidate profiles are provided by the adopted oscillator models. A formulation over a range of wavelengths is computationally more tractable than a formulation at distinct wavelengths. In a discrete approach, the search space increases with the number of wavelengths and it is cumbersome to determine a physically meaningful refractive index (and/or extinction coefficient) profile out of every solution point at each wavelength, while satisfying the associated constraints. However, our proposed optimization approach generates the candidate solutions from a search space restricted by the Kramers-Kronig relation and fits all the given spectral data points simultaneously.

## Data curation

The proposed methodology is implemented on a diverse data set for two kinds of film materials: (A) metal-oxide, and (B) perovskite (MAPbI_3_). Different types of data used in the present study are summarized in Table 1, and the details are described below.

**Table 1:** Obtaining reflectance and transmittance spectra for different data types. The film thickness in experimental data are measured using combinations of the process variables (M, rpm). Further details are available in the Data Curation section.

Type	Optical Constants (*n*, *k*)	Thickness (*d*)	Spectra (*R*, *T*)
Fully-synthetic Data	1116 varieties of (*n*, *k*) profiles simulated	random values within a range: 10 − 2010 nm	calculation method: transfer-matrix
Semi-synthetic Data	18 varieties of (*n*, *k*) profiles from literature [1]	incremental values within a range: 10 − 2010 nm	calculation method: transfer-matrix
Experimental Data	metal-oxides: ITO, NiO perovskite: MAPbI_3_	concentration (M): 0.5, 1.25, 1.5 coating speed (rpm): 3000, 6000	spectrophotometric measurements

*Fully-synthetic dataset:* There involves two steps in preparing the fully-synthetic data: (i) emulate refractive indices *n* and *k* by using a single Tauc-Lorentz oscillator (commonly used for metal oxide materials), and (ii) obtain the reflectance *R* and transmittance *T* by using the transfer-matrix method with the (*n*, *k*) profiles obtained in step (i) and the user-specified *d* values, where the wavelength range selected in the UV-Vis-NIR is 350–1000 nm. For this dataset, 1, 116 (*n*, *k*) profiles were simulated with a python implementation of the one-oscillator Tauc-Lorentz model in step (i), and 10 random thickness values within a range 20 − 2000 nm were used in step (ii) for obtaining (*R*, *T*) spectra.

*Semi-synthetic dataset:* There involves two steps in preparing the semi-synthetic data: (i) obtain refractive indices *n* and *k* from the literature [1] (for perovskite materials); (ii) obtain the reflectance *R* and transmittance *T* by using the transfer-matrix method with the (*n*, *k*) profiles obtained in step (i) and the user-specified *d* values, where the wavelength range selected in the UV-Vis-NIR is 350–1000 nm. For this dataset, a set of 18 distinct (*n*, *k*) profiles were selected from the literature in step (i), and 15, 640 (*R*, *T*) spectra were simulated with a python implementation of the transfer-matrix method by assigning thickness values *d* in a range 10 − 2010 nm with an increment of 1 nm. Unlike the fully-synthetic dataset, the semi-synthetic dataset only requires one-step simulation, i.e., the simulation of (*R*, *T*) spectra using the transfer-matrix method.

*Experimental dataset:* The metal-oxide films, ITO and NiO, are sputtered via physical vapor deposition (PVD) on a glass substrate using an *FHR SV-540* in-line sputtering tool. The MAPbI_3_ perovskite film is deposited on a glass substrate with two process variables affecting thickness, which are the concentration of the perovskite precursor solution (PbI_2_ and MAI with molar ratio of 1:1) and the spin coating speed. We conduct two characterizations: (1) spectrophotometry (UV-Vis) with an *Agilent Cary 7000 UV-Vis-NIR Spectrophotometer* [45], to obtain the optical reflection and transmission, and (2) profilometry with an *KLA Tencor P-16 + Plus Stylus Profiler*, to obtain the thickness of the deposited films.

The extinction coefficient of a material is its characteristic/intrinsic property. In the present study, we have considered a variety of materials, including hypothetical and realistic metal-oxides and MAPbI_3_ perovskites. Each material’s extinction coefficient has a specific maximum and minimum. Considering various material samples used in the current data sets (fully-synthetic, semi-synthetic and experimental), the maximum and the minimum of all the extinction coefficients are 2.0 and 0.00, respectively. Note that the extinction coefficients in the semi-synthetic and experimental data sets are more realistic than that of the fully-synthetic data set, which never go beyond the peak value of 1.5.

## Results

*Implementation Aspects:* During the implementation for extracting different films’ thickness and optical constants, wavelength (λ) is considered as the independent variable. The reflectance and transmittance spectra are measured for a wavelength range of 350 to 1000 nm. The inverse problem of determining *d*, *n*(λ), *k*(λ) from *R*, *T* is not unique. So, there is a risk that an optimization algorithm unravels solutions that do not make sense physically. To mitigate this issue, we pose bounds on the decision variables involved in TLO and GO models such that negative *n* and *k* are always discouraged during the optimization process. The decision variables associated with the TLO ensemble model, i.e. *A*_*i*_, *E*_0*i*_, *C*_*i*_, *E*_*g*_, *ϵ*_∞_, *d*, are selected from a bounded search space: {0, 100}, {0, 10}, {0, 10}, {0, 5}, {0, 2}, {20, 2000}. In TL ensemble model, 2, 3 and 4, 5 oscillator components are chosen for type A and B materials, which gives rise to 9, 12 and 15, 18 variables to be optimized, respectively. For each TLO oscillator, two parameter constraints: |*E*_0*i*_| > *E*_*g*_ and $|E_{0i}|>C_{i}/\sqrt{2}$, are maintained during optimization via penalizing the objective function value. The decision variables associated with the GO model, i.e. *A*_*i*_, *μ*_*i*_, *σ*_*i*_, $\bar{\text{n}}$, *d*, are selected from a bounded space: {0, 5}, {3, 9}, {0.2, 1.1}, {0, 5}, {20, 2000}. In the GO ensemble model, 2, 3 and 4, 5 oscillator components are chosen for type A and B materials, giving rise to 8, 11 and 14, 17 variables to be optimized, respectively. To save function evaluations, a small population size is selected as per CMAES’s default parameter setting [31] that suggests a population of 10 candidates to tackle 8, 9 decision variables, a population of 11 candidates to tackle 11, 12, 14 decision variables and a population of 12 candidates to tackle 15, 17 decision variables in the objective function.

*Algorithm Selection:* We apply two EAs, namely Genetic Algorithm (GA) and Covariance Matrix Adaptation Evolution Strategy (CMAES), for optimizing the parameters associated with the adopted optical dispersion models: TLO ensemble and GO ensemble. To validate the performance of CMAES and GA on TLO as well as GO ensembles, 20 samples are drawn randomly from the fully-synthetic data set. For each sample, three runs are considered with a maximum of 200 iterations and the best result is stored. Table 2 presents a performance comparison between the employed local and global optimization algorithms, i.e. LM vs. GA vs. CMAES. The average loss is drastically higher in case of the local optimization algorithm than the global optimization algorithms because the former drives only a single point that may easily get trapped by local optima whereas the latter drives a population of candidates to efficiently explore the search space while avoiding local traps. Therefore, the performance of LM very much depends on the initial condition; a better starting point leads to a lower loss value. On the contrary, the performance of EAs like GA or CMAES is less sensitive to the initial population. Table 2 shows that the average loss achieved by CMAES is the lowest. This study bolsters the use of CMAES over GA in the underlying inverse problem. Upon selecting CMAES, it is applied onto the fully-synthetic, the semi-synthetic and the experimental data.

**Table 2:** Performance evaluation of local and global optimization algorithms.

Algorithm	LM	GA	CMAES
Model			
TLO	61.6164	3.0407	1.3828
GO	90.5451	2.5997	2.4713

### Performance on synthetic data

For each sample, five runs are considered with a maximum of 1500 iterations and the best result is stored. We cut the run if CMAES reaches a loss value below 0.05 (stopping criteria). A loss value of 0.05 in Eq (8), refers to a mean square error of 0.05/651 = 7.68*e* − 5 for (1000 − 350) + 1 = 651 wavelengths in the given range. A successful occasion is counted if the thickness estimation error is below 10% along with a minimum loss of ≤ 0.17 (saving criteria).

**Fig 2:**
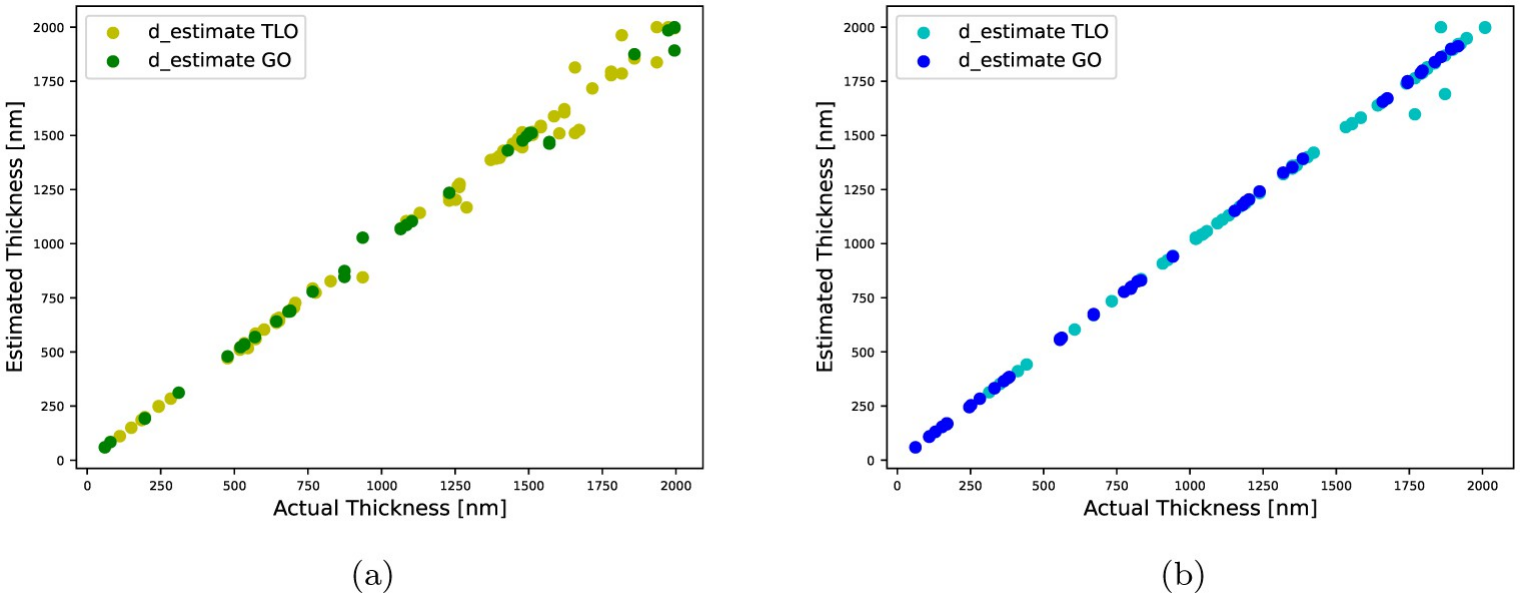
Visual representation of the actual vs. estimated thickness values for the successful occasions reported in Tables 3 and 4: (a) thickness estimates of type A films, (b) thickness estimates of type B films.

**Fig 3:**
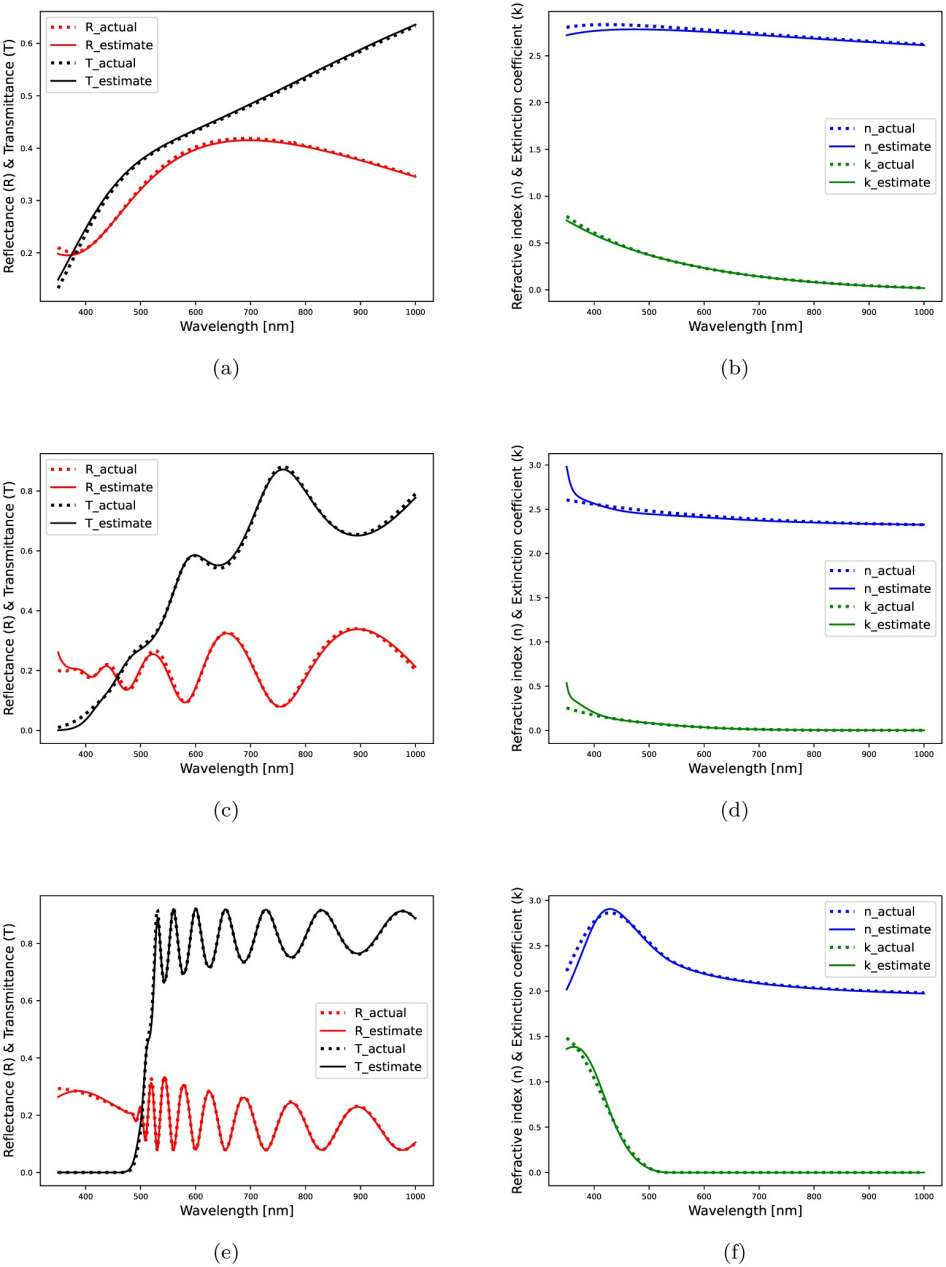
Inverse solutions obtained using the synthetic spectral data of metal-oxide films: (a) Actual and estimated spectra with optimized TLO ensemble; total estimation loss = 0.0260, (b) Actual and estimated optical constants with TLO; actual and estimated thickness = 60.0, 60.26 *nm*, (c) Actual and estimated spectra with optimized GO ensemble; total estimation loss = 0.1011, (d) Actual and estimated optical constants with GO; actual and estimated thickness = 477, 479.71 *nm*, (e) Actual and estimated spectra with optimized TLO ensemble; total estimation loss = 0.0493, (f) Actual and estimated optical constants with TLO; actual and estimated thickness = 1230, 1233.54 *nm*.

*Results using fully-synthetic data:* CMAES is applied onto 100 samples randomly drawn from the entire synthetic dataset, and a detailed performance evaluation is presented in Table 3. Overall, the performance metrics, *EE*_*d*_, *mEE*_*n*_ and *mEE*_*k*_, suggest less variance in the estimated thickness values and more variance in the estimated refractive indices. The model-based optimization prove to be effective with TLO-2 and GO-3. The thickness estimates of various samples for the successful occasions, are shown in Fig 2. For three different inverse solutions, the estimated refractive index (*n*) and extinction coefficient (*k*) are shown in Fig 3, which are in conformity with their references. In Table 3, we mention the medians *mEE*_*n*_ and *mEE*_*k*_ as the associated R^2^-score sequences contain outliers that might skew the average of scores. An outlier refers to an inverse solution where the real or imaginary refractive index estimates have high variances with respect to their reference profiles, although the corresponding thickness estimate is satisfactory.

**Table 3:** Estimation performance of the adopted oscillator ensemble models applied on 100 samples of type A films, picked randomly from the fully-synthetic data set. *EE*_*d*_: R^2^-score between original and estimated thickness values, *mEE*_*n*_: median of R^2^-scores between original and estimated *n*(λ) arrays; *mEE*_*k*_: median of R^2^-scores between original and estimated *k*(λ) arrays; *mEE*_*R*_: mean of R^2^-scores between original and estimated *R*(λ) arrays; *mEE*_*T*_: mean of R^2^-scores between original and estimated *T*(λ) arrays; *SR*: success rate = number of successful occasions (*n*_*ss*_)/ total occasions (*n*_*s*_); and *mFE*: average number of function evaluations: $\left(1/n_{ss}\right)*\sum_{i=1}^{n_{ss}}FE_{i}$; and *mFE* denotes the average number of function evaluations (iterations × population size).

Thin Film	Optical Model	Scores						
		*EE*_*d*_	*mEE*_*n*_	*mEE*_*k*_	*mEE*_*R*_	*mEE*_*T*_	*SR*	*mFE*
type A	TLO -2	0.99502	0.90654	0.99779	0.93573	0.98083	**0.62**	228.32
	TLO -3	0.99500	0.94185	0.99637	0.96052	0.98018	0.57	266.59
	GO -2	0.99884	-1.41127	0.76782	0.94592	0.87954	0.23	294.87
	GO -3	0.99627	0.25544	0.95294	0.96604	0.99185	0.27	558.15

**Fig 4:**
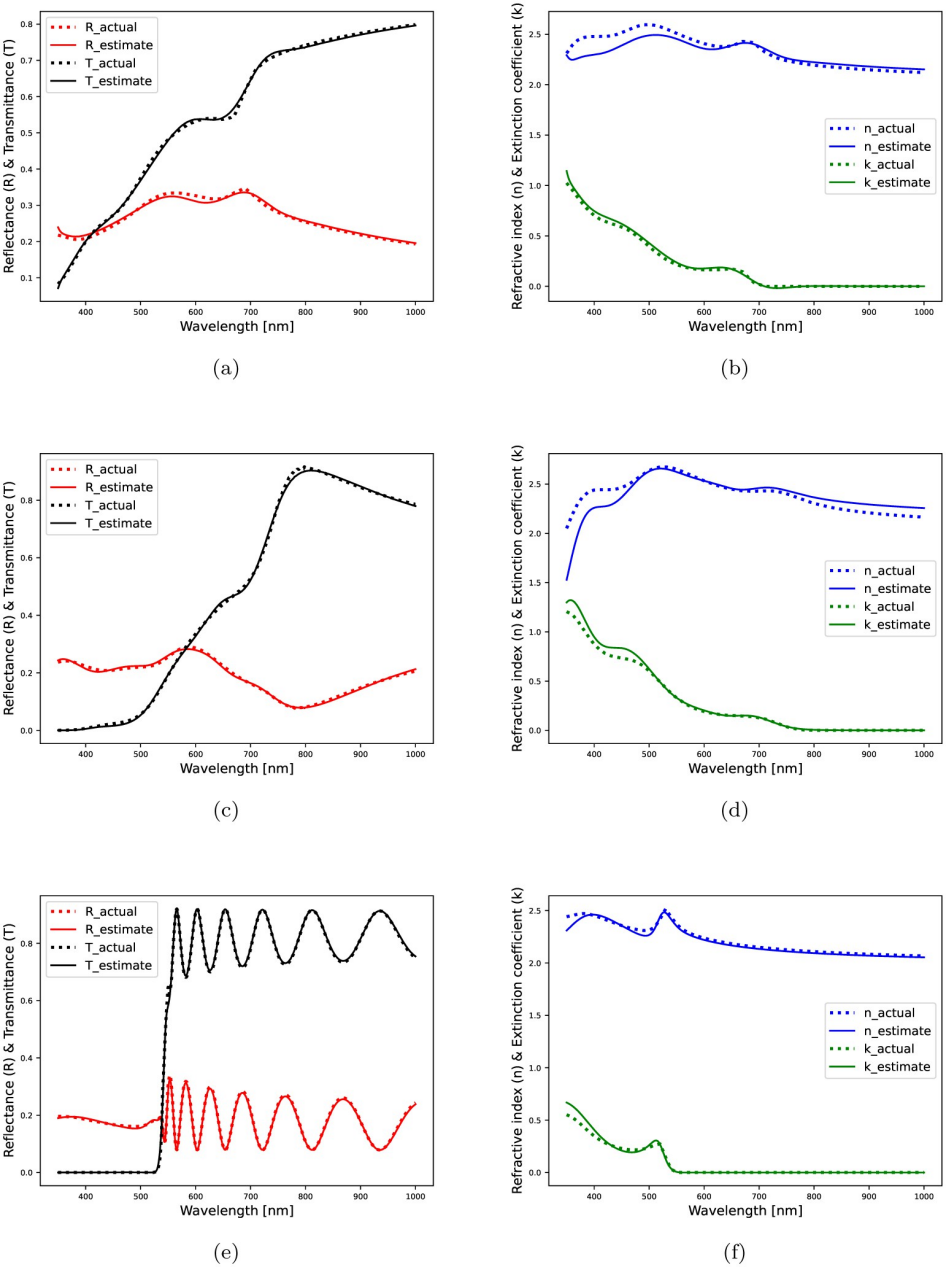
Inverse solutions obtained using the semi-synthetic spectral data of MAPbI3 films: (a) Actual and estimated spectra with optimized GO ensemble; total estimation loss = 0.0530, (b) Actual and estimated optical constants with GO; actual and estimated thickness = 62.0, 59.44 *nm*, (c) Actual and estimated spectra with optimized GO ensemble; total estimation loss = 0.0454, (d) Actual and estimated optical constants with GO; actual and estimated thickness = 169.0, 166.53 *nm*, (e) Actual and estimated spectra with optimized TLO ensemble; total estimation loss = 0.0335, (f) Actual and estimated optical constants with TLO; actual and estimated thickness = 1350.0, 1359.86 *nm*.

*Results using semi-synthetic data:* CMAES is applied onto 100 samples randomly drawn from the semi-synthetic dataset, and a detailed performance evaluation is presented in Table 4. Overall, *EE*_*d*_ is quite good although *mEE*_*n*_ and *mEE*_*k*_ are slightly worse. The model-based optimization prove to be effective with TLO-4 and GO-5. The thickness estimates of various samples for the successful occasions are shown in Fig 2. For three different inverse solutions, the estimated refractive index (*n*) and extinction coefficient (*k*) in Fig 4 exhibit a good agreement with the original profiles.

**Table 4:** Estimation performance of the adopted oscillator ensemble models applied on 100 samples of type B films, picked randomly from the semi-synthetic dataset. Note that the spectral data are in consistent with linear spectrophotometric measurements.

Thin Film	Optical Model	Scores						
		*EE*_*d*_	*mEE*_*n*_	*mEE*_*k*_	*mEE*_*R*_	*mEE*_*T*_	*SR*	*mFE*
type B	TLO -3	0.99528	0.79032	0.93447	0.98572	0.99968	0.53	719.43
	TLO -4	0.99672	0.87354	0.95219	0.98533	0.99968	**0.63**	755.65
	TLO -5	0.99750	0.79227	0.94078	0.98655	0.99969	0.48	747.08
	GO -3	0.99988	-0.17827	0.79189	0.97610	0.99896	0.10	719.7
	GO -4	0.99996	0.30902	0.79027	0.98416	0.99895	0.36	1161.11
	GO -5	0.99997	0.44936	0.87493	0.98771	0.99921	0.43	1321.56

**Fig 5:**
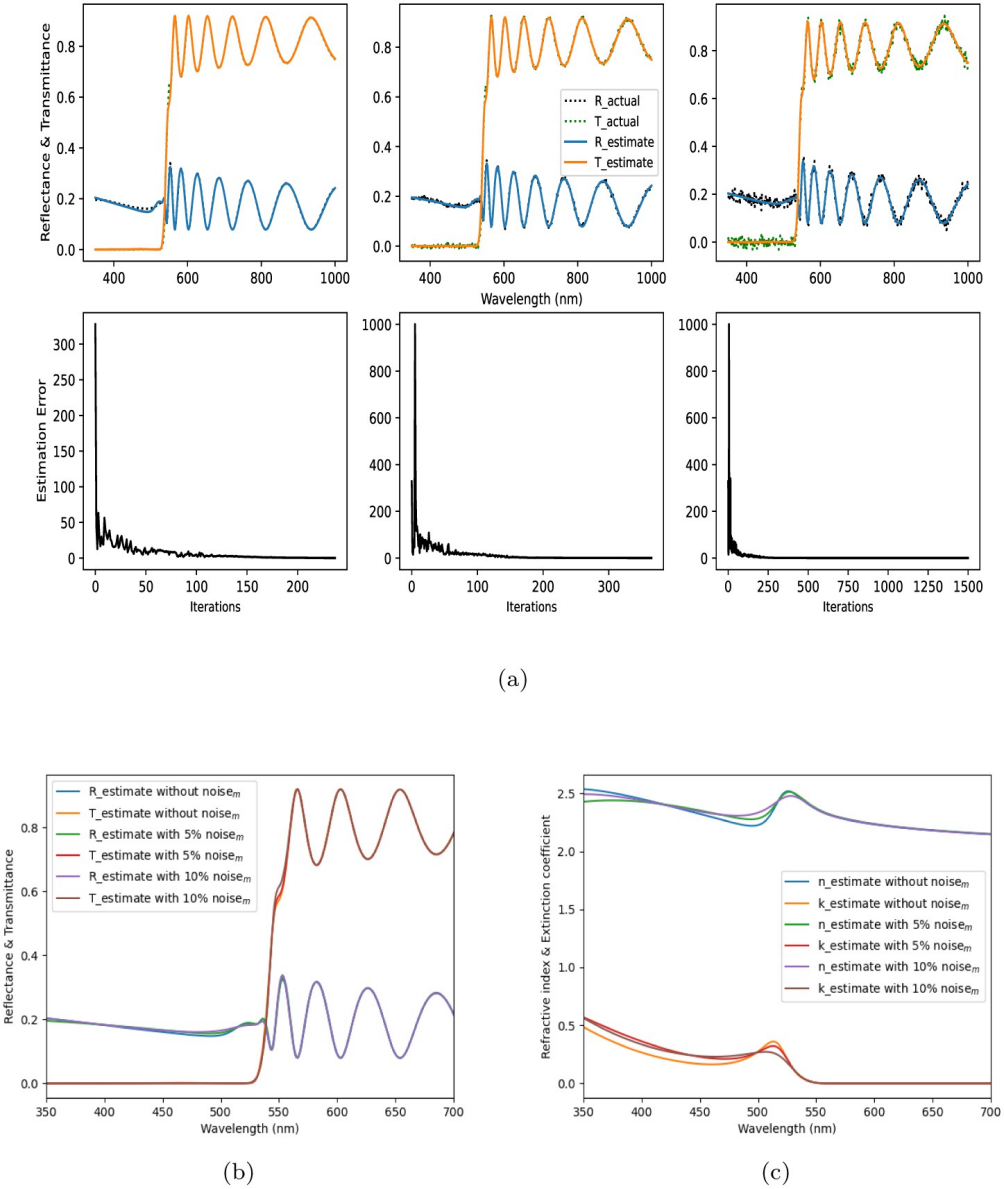
Effect of measurement noise in estimation performance: (a) The top row presents noisy spectral data references and estimates, and the bottom row presents the evolution of estimation errors with iterations; the reference and the estimated reflectance and transmittance are in good agreement with each other. The noise level increases from left to right column-wise as 0%, 5%, 10% noise*_m_*, respectively (b) Estimated spectra vs. wavelength, (c) Estimated optical constants vs. wavelength.

To verify the robustness of the proposed method against measurement uncertainty, we inject normally distributed random disturbances into the reflectance and transmittance data of a *d* = 1350 nm thick perovskite film and run the optimizer to determine the respective inverse solutions. A performance comparison with different levels of measurement noise, i.e. noise_*m*_, is shown in Fig 5. As per Fig 5(a), the number of iterations required by the optimizer increases as the noise level increases from 5% to 10% of the (*R*, *T*) data. Without any noise, the optimizer takes 238 iterations to reach the global minimum with an estimation error <0.05. With 5% noise_*m*_, the optimizer is able to minimize the estimation error below the threshold 0.05 in 367 iterations, however, with 10% noise_*m*_, the error is 0.201 after 1500 iterations. The retrieved thickness estimates are: 1352.63, 1350.33, 1352.26 with 0%, 5%, 10% noise_*m*_, respectively. Due to the presence of noise_*m*_, Fig 5(c) indicates a downward shift in the estimated refractive index and an upward shift in the estimated extinction coefficient for wavelengths below 520 nm. Thus, the proposed method is robust against measurement noise up to certain extent.

### Performance on experimental data

**Fig 6:**
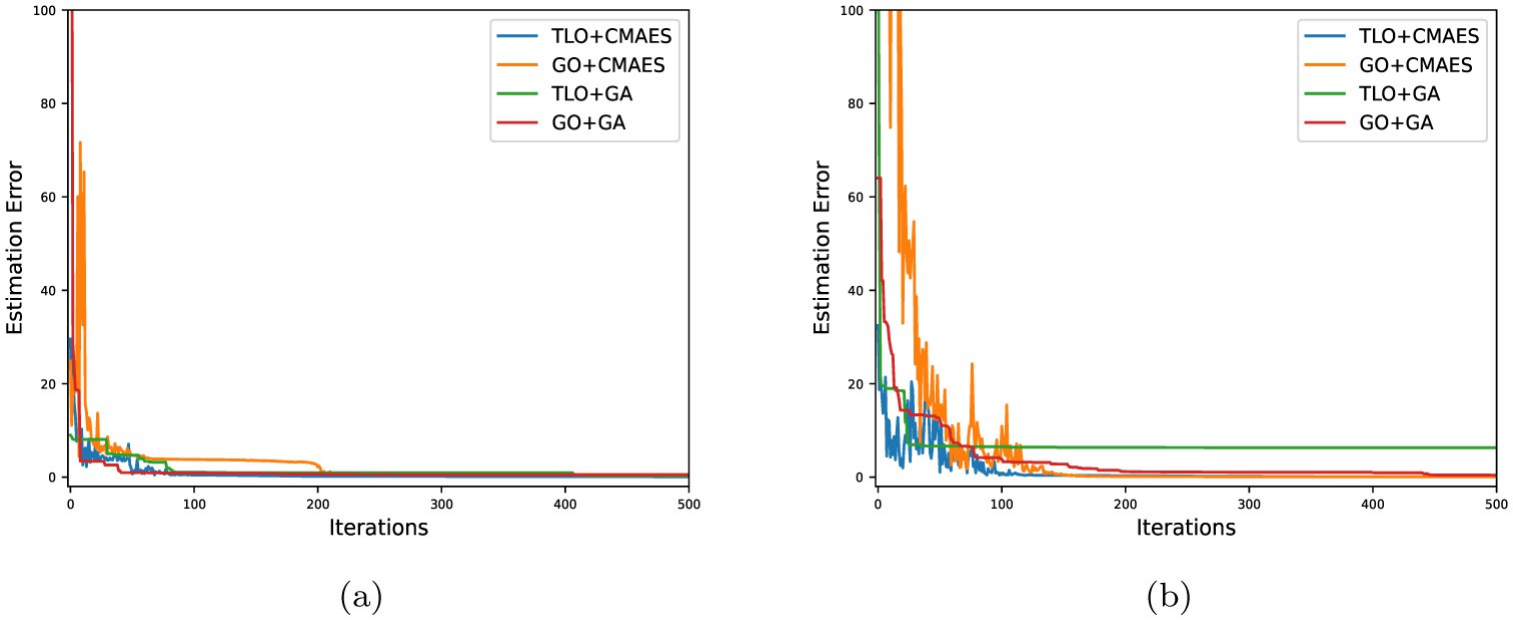
Evolution of estimation errors during model-based optimization with CMAES: (a) Error evolution for film 1, (b) Error evolution for film 2 in Table 5.

**Fig 7:**
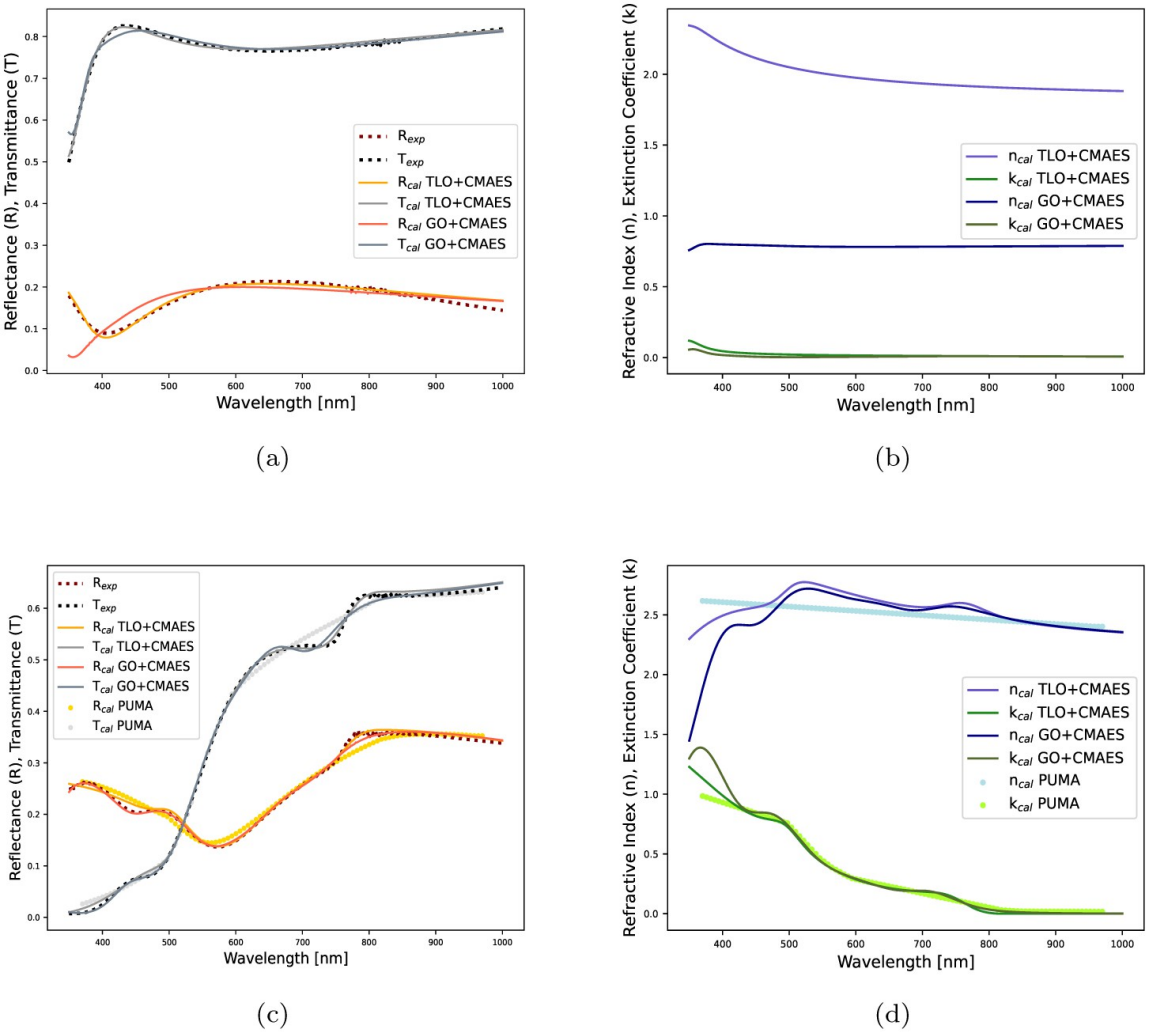
Estimated optical constants from the experimentally measured spectra of two different films: (a) Measured vs. estimated spectra for ITO film, (b) Estimated refractive index and extinction coefficient estimates of ITO film. (c) Measured vs. estimated spectra for MAPbI_3_ film, (d) Estimated refractive index and extinction coefficient estimates of MAPbI_3_ film.

Moreover, the model-based optimization with evolutionary algorithms, GA and CMAES, is used to extract the thickness and the optical constants of a 91 *nm* thick ITO film and a 99 *nm* thick MAPbI_3_ film from the experimentally measured spectra. For each method, the respective optimizers are run ten times and the best results are recorded. The maximum number of allowed iterations is 1000, however, we cut the run if the estimation error goes below 0.05. For two different films, the resulting thickness estimates and the related optimization details are presented in Table 5. The evolution of estimation errors during the optimization process are shown in Fig 6. Even with a small population of 12 candidates, CMAES gives an estimation error of order 10^−2^ in 500 iterations and it reduces faster than that of GA, as supported by Fig 6. For the second film in Table 5, the fitting performance and estimated complex refractive index are shown in Fig 7. In Fig 7(a), TLO fits the spectra better than GO throughout the wavelength range of 350 − 1000 nm. Fig 7(c) help visualizing that GO fits the spectra better than TLO in the wavelength range of 350 − 500 nm, with an appropriate broadening [46]. These results justify that the model-based optimization with CMAES can produce accurate *d*, *n*(λ), *k*(λ) estimates using real data with experimental uncertainty.

**Table 5:** Estimation performance of various methods on experimental data. In the present spectrophotometry, light beams fall straight (normally) on the films.

Film	Method	TLO+CMA	GO+CMA	TLO+GA	GO+GA
	Outcome				
#1 metal-oxide	d (nm)	201.19	91.76	89.33	89.04
	L	0.0611	0.5399	0.2648	0.4363
	iteration	1000	1000	1000	1000
	nFE	10000	11000	10000	11000
#2 perovskite	d (nm)	99.05	99.33	33.05	98.76
	L	0.0499	0.0499	0.2369	0.2417
	iteration	600	556	1000	1000
	nFE	7200	6672	12000	12000

*Point-wise vs. Simultaneous Optimization:* Earlier research [47] introduced a point-wise unconstrained minimization algorithm (PUMA) that considers finite number of points in a range of wavelengths and makes use of repeated calls to solve the underlying estimation problem. For instance, to extract the thickness and the optical constants of a thin-film from the experimental data, reported in Table 5, the following PUMA calls are made recursively.

>puma singl0099b 4 2 10 B 100 0370 0970 3000 1e+100 0 0010 2000 50 0400 950 50 3 5 1 3 5 1 0.10 0.10 0.05

>puma singl0099b 4 2 10 B 100 0370 0970 5000 1.633587e-01 9 0050 0150 01 0400 0700 50

>puma singl0099b 4 2 10 B 100 0370 0970 50000 2.935665e-02 9 0090 0110 01 0450 0650 10

In this case, a quadratic error of 2.9356*e* − 2 with respect to 100 distinct wavelengths (points) in 350 − 1000 nm, is attained by PUMA. The estimated thickness (99 nm) is accurate with reference to the original thickness value (99 nm), though the estimated optical constants in Fig 7(d) do not capture fine transitions and they are not in good agreement with the index profiles found by our proposed method. Moreover, there is a lack of instruction on how to select a range for inflection points in PUMA calls and how many calls are sufficient to solve an inverse problem.

**Fig 8:**
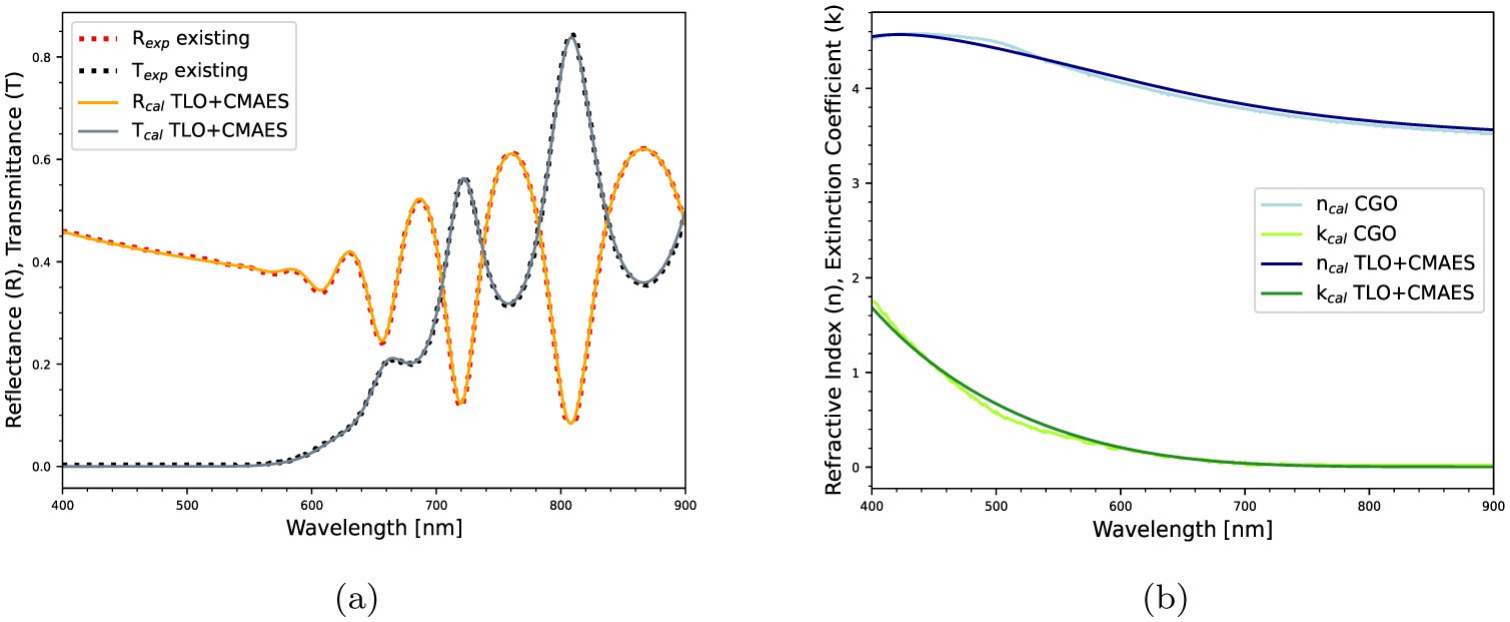
Performance comparison between estimation techniques: (a) Measured vs. estimated spectra, (b) Estimated optical constants.

*Comparison with Existing Package:* Furthermore, the proposed method is applied on a literature data (*R*, *T*) to extract the optical constants and the thickness of a Si film [15]. In this case, a population of 50 candidates is leveraged and a minimum loss of 0.02 is selected as the stopping criteria for the CMAES algorithm. The optimal solution is presented in Table 6 and the estimated optical constants are shown in Fig 8. The benefits of the proposed method over the existing Optichar Software (OS) and Clustering Global Optimization (CGO) algorithm, are as follows: (i) In OS, a user has many options (normal, arbitrary dispersion, Sellmeier) for a model-based iterative optimization procedure, however, there are no information available on the decision variables and the optimization algorithm. The proposed method offers clarity on the optimization algorithm and the associated variables; (ii) The effectiveness of CGO depends on the choice of initial intervals of the associated decision variables [35]. It is difficult to explore all the minima with a small initial interval, and on the other hand, a broad initial interval might leave a sharp minimum undetected. Unlike CGO, the CMAES algorithm’s performance is not so sensitive to its parameter bounds and the choice of bounds can be made without requiring much domain knowledge, as shown in Table 6; (iii) Also, CMAES performs faster than CGO and OS as it minimizes the error function with less evaluations (FE), as reported in Table 6.

**Table 6:** Performance comparison between the proposed method and the existing methods based on OptiChar software and CGO algorithm. Here, the estimation error is defined according to the reference [15]. Note that the ^§^ error is calculated with reference to the spectral data [15] generated using a plot digitizer [48], while considering 651 points in the wavelength range of 400 − 900 *nm*.

Method	Bounds	Solution	Error [15]	*n*	*k*	FE
OS	×	667.1	0.647	4.427 at 500 nm	0.395 at 500 nm	×
				3.809 at 700 nm	0.040 at 700 nm	
CGO	*A*_1_: 70 − 76	73.364	0.378	4.487 at 500 nm	0.581 at 500 nm	10^5^
	*A*_2_: 6.5 − 12.5	9.406		3.789 at 700 nm	0.040 at 700 nm	
	*A*_3_: 0.4 − 1.4	0.832				
	*E*_01_: 3.36 − 3.96*eV*	3.664				
	*E*_02_: −2.24 − 1.64*eV*	−1.933				
	*E*_03_: −2.74 − 2.14*eV*	−2.425				
	*C*_1_: 1.7 − 2.3	1.986				
	*C*_2_: 1.15 − 1.75	1.448				
	*C*_3_: 0.15 − 0.75	0.429				
	*E*_*g*_: 0.7 − 0.9*eV*	0.842				
	*ϵ*_∞_: 2.28 − 2.88	2.562				
	*d*: 660 − 680*nm*	670.5				
Proposed	*A*_1_: 0 − 100	13.729	^§^0.004	4.423 at 500 nm	0.665 at 500 nm	551 × 50 = 27550
	*A*_2_: 0 − 100	96.837		3.829 at 700 nm	0.039 at 700 nm	
	*A*_3_: 0 − 100	31.035				
	*E*_01_: −5 − 5*eV*	−1.953				
	*E*_02_: −5 − 5*eV*	3.810				
	*E*_03_: −5 − 5*eV*	4.714				
	*C*_1_: 0 − 10	1.549				
	*C*_2_: 0 − 10	2.418				
	*C*_3_: 0 − 10	2.504				
	*E*_*g*_: 0 − 5*eV*	0.960				
	*ϵ*_∞_: 0 − 5	1.526				
	*d*: 20 − 2000*nm*	664.4				

### Analysis and discussion

In Fig 2(a) and 2(b), the optimized TLO ensemble model finds more number of accurate thickness estimates (within 10% of the original values) than the optimized GO ensemble model, which is also supported by Table 3. The thickness estimates are slightly dispersed around their original values for films with *d* > 1.5 *μm*. At higher film thickness, the optical constants exhibit a nonlinear dependence commonly observed in earlier research findings [49]. In Fig 3(b), *n* and *k* estimates exactly match with the original profiles throughout, however, in Figs 3(d), 4(b) and 4(d), there exist slight differences between the original and estimated *n*, *k* profiles especially in the low-wavelength regime partially including ultraviolet-visible spectral range. To further inspect this quantitatively, we split the entire wavelength range into two sectors and highlight the estimation performance of the well fitted models as per Tables 3 and 4.

- TLO-2 for type A films: For a wavelength range of 350 − 500 nm, the estimation metrics are *mEE*_*n*_ = 0.41617, *mEE*_*k*_ = 0.98999; and for a wavelength range of 500 − 1000 nm, the estimation metrics are *mEE*_*n*_ = 0.83078, *mEE*_*k*_ = 0.99870.
- TLO-4 for type B films: For a wavelength range of 350 − 500 nm, the estimation metrics are *mEE*_*n*_ = −0.47954, *mEE*_*k*_ = 0.72331; and for a wavelength range of 500 − 1000 nm, the estimation metrics are *mEE*_*n*_ = 0.97758, *mEE*_*k*_ = 0.95517.

The above reported results reveal that the fitting error in *k*(λ) gets amplified into *n*(λ) as it propagates through the Kramers-Kronig integration. In the low-wavelength regime, *mEE*_*n*_ and *mEE*_*k*_ deteriorate for perovskite materials, which could be due to the interaction between multiple inter-band optical transitions [50] or film inhomogeneity [4].

The error metrics reported in Tables 3 and 4 reveal the challenge in achieving a good accuracy of *n*(λ), *k*(λ) estimates simultaneously with *d*. The achieved results justify that the inter-band transitions for metal-oxide and perovskite materials are well captured by Tauc-Lorentz oscillators. Moreover, the model-based optimization with CMAES can handle measurement uncertainty and extract optical parameters from experimental data, as supported by Table 5 and Fig 7. It is worth noting that a local search algorithm, such as sequential least square programming (SLSQP from ‘*scipy.optimize*’), can be applied to the estimates found by an EA to further improve the solution accuracy if needed.

Inverse problems involve one-to-many mappings and often they are ill-posed [38, 51], therefore, finding exact solutions to such problems is challenging. Further, the difficulty level rises when the inverse solutions are extracted from noisy measurements. The present work demonstrates that the proposed model-based optimization with CMAES does a reasonably good job in extracting accurate inverse solutions. The nature of complex refractive index profiles varies across diverse materials, such as inorganic, organic, and other miscellaneous materials. Thus, the number of oscillator components to be used in an optical ensemble model depends on the type of the concerned material(s), and it is difficult to come up with an universal rule of selecting the same. The current choice is subjective to two types of film materials: metal-oxide and perovskite. To tackle multiple optical transitions in case of MAPbI_3_ perovskite films, the number of oscillator components is selected as higher than that of metal-oxides films. In general, our solver application can be extended to a variety of materials by adjusting the number of composition elements in the optical dispersion models. The proposed evolutionary optimization approach does not require any prior learning or memory-based mapping. The successful runs take only about a few minutes to find an inverse solution with an i7-4600M CPU@2.90 GHz processor. The present approach utilizes the spectral data at just one incident angle of light, however, in future, additional spectral information (at multiple incident angles) can be considered to alleviate the effect of uncertainty in the experimental measurements.

## Conclusion

The proposed model-based optimization with covariance matrix adaptation evolution strategy (CMAES) succeeds in extracting the thickness and the optical constants of metal-oxide and perovskite films from spectrophotometric data using tangible parameters. The employed evolutionary algorithm, CMAES, proves to be efficient in finding globally optimal solutions without spending much function evaluations, and it is robust against measurement uncertainties as well. Overall, the proposed method finds accurate thickness estimates and it can estimate complex refractive indices with multiple transitions.
